# Congenital coronary artery fistula in pediatric patients: transcatheter versus surgical closure

**DOI:** 10.1186/s12872-020-01769-7

**Published:** 2020-11-16

**Authors:** Xiaoyong Wang, Chengcheng Pang, Xiaobing Liu, Shushui Wang, Zhiwei Zhang, Jimei Chen, Jian Zhuang, Chengbin Zhou

**Affiliations:** 1 The First Affiliated Hospital of Shantou University Medical College, Shantou, China; 2 Department of Cardiovascular Surgery, Guangdong Provincial Cardiovascular Institute, Guangdong Provincial Key Laboratory of South China Structural Heart Disease, Guangdong Provincial People’s Hospital, Guangdong Academy of Medical Sciences, No. 106, Zhongshan 2nd Road, Yuexiu District, Guangzhou, 510000 China; 3 Department of Pediatric Cardiology, Guangdong Provincial Cardiovascular Institute, Guangdong Provincial People’s Hospital, Guangdong Provincial Academy of Medicine, Guangzhou, China

**Keywords:** Fistula, Congenital heart disease, Coronary artery disease

## Abstract

**Objectives:**

Transcatheter closure (TCC) and surgical closure (SC) are the two main approaches for congenital coronary artery fistula (CCAF), but data on the comparisons of the efficacy and safety of these two approaches are limited.

**Methods:**

We retrospectively reviewed pediatric patients with CCAF in Guangdong Cardiovascular Institute between January 2002 and December 2017. Patients who were qualified into our criteria were included into final analysis. The rate of successful closure and complications during hospitalization and at follow-up were compared between SC and TCC groups.

**Results:**

In total, 121 pediatric patients (male, n = 69; female, n = 52) with CCAF were divided to TCC (n = 63) and SC groups (n = 58) according to the indications. The mean age was 5.3 ± 1.4 years. The baseline characteristics of these two groups were similar except for the fistula anatomic feature. After adjusted for the fistula anatomy, compared to SC, TCC was associated with higher risk of major complications (p = 0.013). Proportions of patients requiring blood transfusion and intra-operative blood loss were higher in SC versus TCC groups, as were longer duration of hospital and ICU stay during hospitalization. In contrast, myocardial ischemia (10.2% vs 0.0%, p = 0.028), residual shunts (16.9% vs 3.6%, p = 0.045) and new-onset moderate-to-severe valve regurgitation (11.9% vs 0.0%, p = 0.013) were higher in TCC group versus SC groups during follow-up.

**Conclusions:**

TCC has less invasive and faster recovery. However, SC had a higher successful rate and lower risk of major complications in pediatric patients.

## Introduction

Congenital coronary artery fistula (CCAF) is a rare congenital anomaly with a connection between coronary arteries and a cardiac chamber or intrathoracic great vessel. Some reports have shown that CCAF was detected in approximately 0.2–0.6% of patients undergoing angiographic examination, and the overall incidence in the general populations is 0.002% [1, 2]. Although CCAF patients are usually asymptomatic [3], a variety of clinical symptoms and cardiovascular complications, including dyspnea, angina pectoris, arrhythmia, bacterial endocarditis and sudden death, can develop secondary to the continuous left-to-right shunting hemodynamic changes. In addition, CCAF with a left-to-left shunt increases the risk of left heart volume overload [4, 5].

Surgical closure (SC) and transcatheter closure (TCC) are the two main approaches for CCAF therapy. Compared to TCC, SC has a historically higher successful rate for fistula closure. Nonetheless, SC requires cardiopulmonary bypass and median sternotomy, and these invasive procedures are associated with increased risk of complications such as infection and bleeding [6–8]. Reidy et al. [9] reported on the first successful case of CCAF closure using TCC in 1983. TCC is less invasive and patients recover from intervention more rapidly [6]. Nonetheless, TCC also has procedures-related complications, including arrhythmia, myocardial ischemia and valves injury. Currently, few studies have compared the efficacy and safety of these two procedures for CCAF therapy in pediatric patients. Therefore, the aim of our current study was to evaluate and compare the successful rate and mid-term of procedure-related complications of these two procedures for CCAF therapy.

## Patients and methods

### Patient selection

We retrospectively reviewed the medical records of all pediatric patients with CCAF receiving therapy in Guangdong Cardiovascular Institute (Guangdong, China) from January 2002 to December 2017. The inclusion criteria were as follows: (1) < 18 years old; and (2) data on in-hospital examination and therapy were available during hospitalization. The exclusion criteria were as follows: (1) asymptomatic patient with small fistula size (< 2 mm); (2) with concomitant other congenital cardiac abnormalities; (3) prior cardiac surgery history; (4) with coexistent coronary artery diseases or severe comorbidities (e.g. Marfan syndrome); and (5) iatrogenic or traumatic coronary artery fistula.

CCAF diagnosis was confirmed by preoperative transthoracic echocardiography (TTE), coronary computed tomographic angiography (CCTA) or selective coronary angiography (SCAG) as appropriate. CCTA or SCAG was performed if TTE was unable to provide a clear depiction of the fistula anatomy. All patients received routine clinical examination including standard 12-lead electrocardiogram, chest X-ray, TTE and laboratory tests. Selection of TCC for isolated CCAF was dependent on two key factors: optimal occlusion site and applicable catheter and device [10, 11]. The optimal occlusion site referred to single, non-tortuous fistula, narrow drainage and the absence of coronary artery branches adjacent to the optimal plugging position or drainage. The applicable catheter and device depended on the ability to cannulate safely the distal site of fistula and the applicable closure device to fistula. If these two key factors were not satisfied, surgical closure will be considered. An informed consent form was obtained from guardians before CCAF closure was performed. The study protocol was approved by the Research Ethics Committee of Guangdong Provincial People’s Hospital, Guangdong Academy of Medical Sciences on March 29, 2018 (No. GDREC 2018186H).

## Procedures

### Surgical closure procedure

In SC group (n = 58), 8 (13.8%) patients were diagnosed and measured by CCTA, 43 (74.1%) patients by SCAG, and 7(12.1%) patients by TTE. After general anesthesia, all patients in the SC group underwent median sternotomy. CCAF was identified by the presence of palpable vibrations and expansion at the fistula site. Aortic and bicaval cannulation was used for cardiopulmonary bypass. CCAF was closed by epicardial or endocardial correction. In 22(37.9%) patients, surgical correction of proximal region of the fistula would be performed if the fistula and coronary artery was enlarged exceedingly. In 29(50.0%) patients, the fistula drainage was completely closed. In 3(5.2%) patients, the fistula was closed by simple ligation, and another 4(6.9%) patients were treated by closing proximal and distal orifices. No anti-platelet or anticoagulation therapy was used after surgery [6, 12].

### Transcatheter closure procedure

**Fig. 1:**
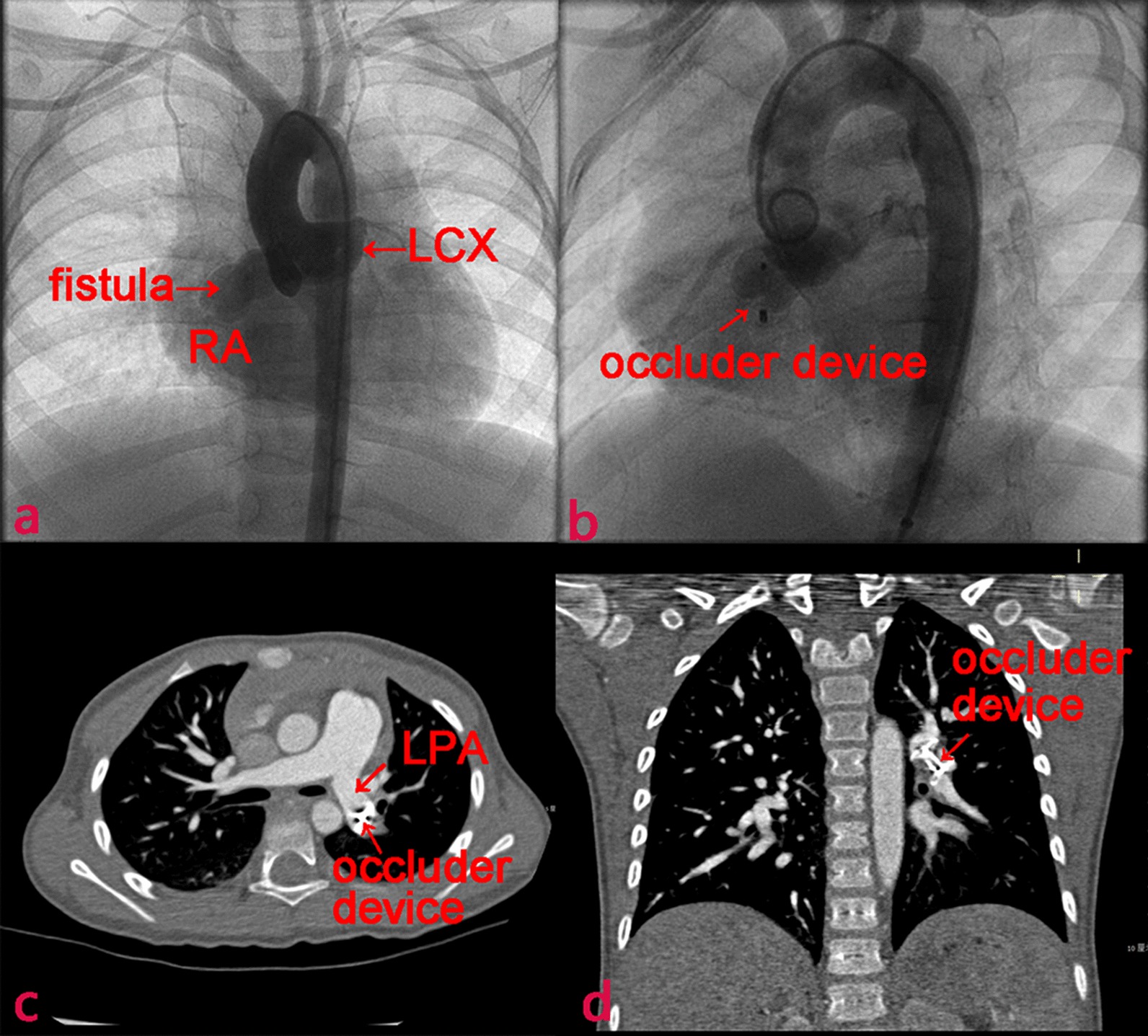
A VSD occluder displaced in a 3 years old child with CCAF (Case No.5). **a** Coronary angiogram showed a coronary artery fistula originating from the LCX and draining into the RA. **b** Fistula closured with aAmplatzer occluder (arrow). **c**, **d** The occluder translocated to the LPA 6 days after implantation. *LCX* left circumflex, *RA* right atrium, *LPA* left pulmonary artery

In TCC group (n = 63), all patients were diagnosed and measured by SCAG, and 57 (90.5%) received TCC procedure under general anesthesia and 6 (9.5%) patients received local anesthesia. Heparin was infused intravenously at a dose of 100 units per kilogram of body weight. Femoral artery and/or femoral vein were used as access site. The anatomic and hemodynamic data of CCAF was evaluated by aortic root angiography. Fistula course, coronary artery branches and drainage were evaluated with multiple views as presented in Fig. 1a. The approach and occluder devices used for fistula closure depended on fistula size and tortuosity, the capability of accessing catheter to distal CCAF and the presence of adjacent coronary branches. An antegrade venous approach was used in 10 patients (15.9%) with CCAF with extremely long and tortuous course. Retrograde arterial approach was used in 4 patients (6.3%) with small-to-medium size of CCAF, and arterio-arterial loop approach was used in 3 patients (4.8%) with medium-to-large caliber CAF draining to the left-sided heart, and 46 (73.0%) used arterio-venous loop approach for medium-to-large CCAF draining into the right-sided heart. A range of occluder devices were used, including 19 (30.2%) Duct Occluders (Lifetech Scientific, Shenzhen, China), 12 (19.0%) Muscular Ventricular Septal Occluders (Lifetech Scientific), 22 (34.9%) Vascular Plugs (Lifetech Scientific) and 10 (15.9%) Cook Coils (William Cook Europe, Bjaeverskov, Denmark). Catheter sheath was detached when it was placed in the appropriate anatomic location (Fig. 1b). Then, angiography was performed to confirm no coronary artery was compromised. Patients were treated with clopidogrel (1 mg/kg, once daily) for at least 1 month and aspirin (3–5 mg/kg, once daily) for at least 6 months after CCAF closure [13, 14].

### Comparisons of outcomes during hospitalization and at follow-up

Data on medical history, physical examination, laboratory examination, radiography reports, electrocardiogram, TTE, catheterization images and surgical notes were extracted from In-patient Management Information System. After closure, patients were followed from the day of intervention until death or end of study on December 31, 2017. At least three examinations of TTE and electrocardiogram were performed at the 3rd, 6th and 12th month after discharge, and then annually thereafter.

The major complications were the rates of re-intervention, postoperative infective endocarditis, medium-sized or above residual fistula, coronary artery thrombosis, ischemic changes on electrocardiogram, and new-onset moderate or severe valvular regurgitation. The definition of medium-sized or above residual fistula was a ≥ 2 mm shunt in TTE during follow-up [13]. New-onset moderate or severe valvular regurgitation was defined using TTE during follow-up [15]. Both major complications and first occurrence time were recorded. Data were collected by two researchers and uncertainty data were consulted with the third independent researcher.

### Statistical analysis

Continuous variables were expressed as mean ± standard deviation or median (interquartile range, IQR) as appropriate. Categorical data were presented as number and percentage. Continuous variables with normal distribution were compared using Student’s t-test, and categorical variables were compared using Chi-square test or Fisher's exact test.

This study was analyzed as intention-to-treat. The Cox proportional hazards regression models was used to examine time to the major complications for all available follow-up patients, of using hazard ratios (HR) and 95% confidence intervals (CI). Baseline variables that were considered clinically relevant with major complications were entered into multivariate Cox proportional-hazards regression model. Candidate variables with a p value < 0.1 on univariate analysis were included in multivariable model. Statistical tests were considered significant when p < 0.05. All statistical analyses were performed using SPSS software version 25.0 (IBM Statistics, USA).

## Results

Of the 121 pediatric patients undergoing CCAF closure between January 2002 and December 2017, 63(52.1%) underwent TCC, and 58(47.9%) underwent SC. 114 (94.2%) patients completed at least 1 year follow-up and the median follow-up duration was 6.9 years (IQR 3.4–10.6 years).

### Baseline characteristics

Baseline characteristics were presented in Table 1. No significant between-group differences in age, gender, body weight, the proportion of patients with clinical symptoms and signs, fistula origin, fistula style, maximum diameter and visible thrombus were observed. However, the minimum diameter of fistula was significantly larger in SC versus TCC groups (4.7 mm vs 3.5 mm; p = 0.003). The percentage of drainage to right atrium (34.5% vs 82.5%) and right ventricle (55.2% vs 9.5%) was significantly different between these two groups (p < 0.001).

**Table 1:** Comparison of baseline characteristics between SC and TCC groups

Characteristic	SC (n = 58)	TCC (n = 63)	p value
Age, year*	5.3 (0.75–17.8)	3.5 (0.5–17.0)	0.732
Male, n (%)	35 (60.3)	34 (54.0)	0.479
Body weight, kg*	17.0 (7.0–55.0)	14.5 (5.5–64.0)	0.601
Symptomatic patients, n (%)	9 (15.5)	5 (7.9)	0.193
*Clinical signs*			
NYHA I-II, n (%)	51 (87.9)	59 (93.7)	0.437
NYHA III-IV, n (%)	7 (12.1)	4 (6.3)	0.437
Cardiac murmur, n (%)	56 (96.6)	60 (95.2)	1
ECG abnormality^a^, n (%)	23 (39.6)	19 (30.2)	0.273
CXR abnormality^b^, n (%)	12 (20.7)	9 (14.3)	0.353
Pre-operation LVEF, %*	70 (61–88)	70 (59–83)	0.335
*Fistula anatomic feature*			
Fistula origin			
Right coronary artery, n (%)	30 (51.7)	41 (65.1)	0.468
Left main coronary artery, n (%)	16 (27.6)	11 (17.5)	
Left anterior descending, n (%)	5 (8.6)	4 (6.3)	
Circumflex, n (%)	7 (12.1)	7 (11.1)	
Fistula drainage			
Right atrium, n (%)	20 (34.5)	52 (82.5)	< 0.001
Right ventricle, n (%)	32 (55.2)	6 (9.5)	
Left atrium, n (%)	0	1 (1.6)	
Left ventricle, n (%)	6 (10.3)	3 (4.8)	
Superior vena cava, n (%)	0 (0.0)	1 (1.6)	
Minimum diameter, mm*	4.7 (2.0–12.0)	3.5 (1.6–10.0)	0.003
Maximum diameter, mm*	7.0 (4.0–15.0)	7.0 (4.0–14.0)	0.068
Fistula type			
Proximal, n (%)	23 (39.7)	36 (57.1)	0.055
Distal, n (%)	35 (60.3)	27 (42.9)	
Presence of visible thrombus, n (%)	1 (1.7)	0	0.479

*SC* surgical closure, *TCC* transcatheter closure, *NYHA* New York Heart Association, *ECG* electrocardiogram, *CXR* chest X-ray, *LVEF* left ventricular ejection fraction

^*^Presented as median (range)

^a^Atrial flutter/fibrillation, right bundle branch block, T wave inversion > 2 leads, ST segment depression > 0.5 mm at least 2 leads, premature atrial or ventricular complex

^b^Cardiac enlargement and/or increased pulmonary vasculature

### Comparison of clinical outcomes during hospitalization

As presented in Table 2, compared to SC group, intra-operative blood loss (19.7 ± 12.8 mL vs 109.7 ± 66.6 mL; p < 0.001) and the proportion of patients requiring blood transfusion were significantly lower (3.2% vs 58.6%; p < 0.001) in the TCC group, as was shorter duration of hospital stay (7.8 ± 4.0 days vs 14.2 ± 4.4 days; p < 0.001). TCC group was not required to perform aortic occlusion and cardiopulmonary bypass and transfer to intensive care unit stay. There was no significant difference in re-intervention and death cases between the two groups. Only 1 patient with CCAF (from RCA to RA) died in the TCC group. This patient developed third-degree atrioventricular block and hypotension after implantation of the occluder. The occluder was immediately removed and cardiopulmonary resuscitation was performed immediately. However, the patient remained died due to the fatal arrhythmia.

**Table 2:** Comparison of clinical outcomes between SC and TCC groups during hospitalization

Variable	SC (n = 58)	TCC (n = 63)	p value
Intra-operative blood loss, mL	109.7 ± 66.6	19.7 ± 12.8	< 0.001
Intra-operative blood transfusion, n (%)	34 (58.6)	2 (3.2)	< 0.001
Hospital stay, days	14.2 ± 4.4	7.8 ± 4.0	< 0.001
Intensive care unit stay, h	16.8 ± 7.2	0.0 ± 0.0	< 0.001
Re-intervention, (%)	0	5 (7.9)	0.058
Death, n (%)	0	1 (1.6)	1

### Comparison of major complications at follow-up

**Fig. 2:**
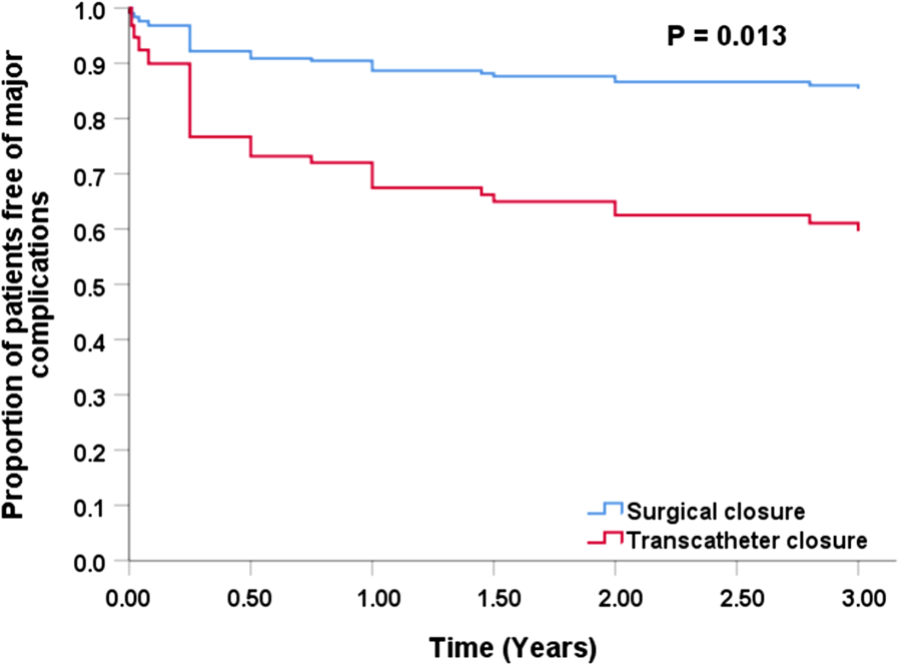
Proportion of patients free of major complications after CCAF

Of Cox regression analysis, SC was associated with lower major complications (Fig. 2). The number of patients with major complications was 8 of 55 (14.5%) in the SC group and 20 of 58 (34.5%) in the transcatheter group after 1-year follow-up (p = 0.014). The unadjusted HR was 2.474 (p = 0.016) and the adjusted HR was 3.272 (p = 0.013) after adjusting for fistula drainage (p = 0.092), fistula type (p = 0.401), minimum diameter of fistula (p = 0.484) and maximum diameter of fistula (p = 0.871) (Table 3).

**Table 3:** Cox proportional hazards regression analysis for major complications

Variables	Univariate			Multivariate		
	HR	95% CI	p value	HR	95% CI	p value
Proximal	Reference					
Distal	0.748	0.380–1.472	0.401	–	–	–
Minimum diameter of fistula	1.063	0.895–1.263	0.484	–	–	–
Maximum diameter of fistula	0.984	0.812–1.193	0.871	–	–	–
Right atrium	Reference					
Right ventricle	0.782	0.358–1.707	0.537	1.693	0.632–4.539	0.295
Left ventricle	1.137	0.339–3.814	0.835	1.552	0.456–5.279	0.482
Left atrium	18.266	2.166–154.024	0.008	14.819	1.753–125.250	0.013
Surgical closure	Reference					
Transcatheter closure	2.474	1.182–5.178	0.016	3.272	1.281–8.357	0.013

*HR* hazard ratio, *CI* confidence interval

As presented in Table 4, compared to the SC group, the rates of residual fistula (16.9% vs 3.6%; p = 0.045) were significantly higher in TCC group, as was new-onset moderate-to-severe valve regurgitation (11.9% vs 0.0%; p = 0.013) and ischemic change on electrocardiogram (10.2% vs 0.0%; p = 0.028). There was no statistically significant difference in the re-intervention, coronary artery thrombosis and infective endocarditis between the SC and TCC groups. No patient died during follow-up.

**Table 4:** Comparison of outcomes between SC and TCC groups at follow-up

Variables	SC (n = 55)	TCC (n = 59)	p value
Residual shunt, n (%)	2 (3.6)	10 (16.9)	0.045
Re-intervention, n (%)	0	4 (6.8)	0.119
Coronary artery thrombosis, n (%)	5 (9.1)	3 (5.1)	0.638
Infective endocarditis, n (%)	1 (1.8)	2 (3.4)	1
New-onset moderate or severe valve regurgitation	0	7 (11.9)	0.013
Tricuspid valve, n (%)	0	5 (8.5)	0.058
Aortic valve, n (%)	0	2 (3.4)	0.496
Ischemic changes on electrocardiogram, n (%)	0	6 (10.2)	0.028

### Clinical characteristics of patients underwent re-intervention

There were 5 re-intervention cases during hospitalization and 4 re-intervention cases at follow-up. Clinical characteristics of patients undergoing re-intervention were summarized in Table 5. There were three reasons for re-intervention: coronary artery branches compromise, fistula re-canalization and valves injury. For example, in case No.5, the occluder migrated and embolized left pulmonary artery 6 days post-TCC (Fig. 1).

**Table 5:** Clinical characteristics of nine patients underwent re-intervention

Characteristic	Patient no								
	1	2	3	4	5	6	7	8	9
Age (years)	1.1	5.7	17.0	6.8	3.1	5.5	3.3	3.0	2.8
Course	RCA → RV	LCX → RA	RCA → RV	RCA → LA	LCX → RA	LMCA → RV	LCX → RA	RCA → RV	RCA → RV
Type	Proximal	Proximal	Proximal	Distal	Proximal	Proximal	Distal	Distal	Distal
Qp/Qs	1.65	1.60	1.74	1.54	1.43	1.70	2.00	1.60	1.45
Catheter approach	A-V loop	A-V loop	Antegrade	Antegrade	A-V loop	A-V loop	A-V loop	A-V loop	A-V loop
Device used	AVP	VSD occluder	VSD occluder	PDA occluder	VSD occluder	PDA occluder	Coil	PDA occluder	PDA occluder
Re-intervention time post-closure	0.2 days	3.0 days	17.0 days	13.0 days	6.0 days	536.0 days	549.0 days	6.0 days	5.0 days

Reasons for re-intervention for each patient: (1) postoperative myocardial ischemia; (2) large residual fistula; (3) adjacent normal coronary artery branch at 2 mm; (4) intra-operative movable occluder; (5) postoperative occluder embolization and tricuspid valve pro-lapse; (6) postoperative myocardial ischemia and aortic valve pro-lapse; (7) postoperative myocardial ischemia and severe aortic valve regurgitation; (8) intra-operative myocardial ischemia; (9) adjacent normal coronary artery branch at 3 mm

*RCA* right coronary artery, *LMCA* left main coronary artery, *LCX* left circumflex, *RA* right atrium, *RV* right ventricular, *LA* left atrium, *AVP* Amplatzer vascular plug, *A-V loop* arterio-venous wire loop, *VSD* ventricular septal defect, *PDA* patent ductus arteriosus

## Discussion

The incidence of CCAF is increasing gradually, which is partly due to a broader use of echocardiography and other imaging modalities [4]. Previously, a common view was that asymptomatic patients with small CCAF has the potential to spontaneous closure and did not require intervention [16]. To those requiring therapy, SC has been recognized as the “gold standard” approach [17, 18]. Nonetheless, with the advancement of catheter equipment and related technologies, TCC for CCAF therapy is increasing use in recent decades, and TCC has been considered as an important alternative to SC [13, 19–21].

**Fig. 3:**
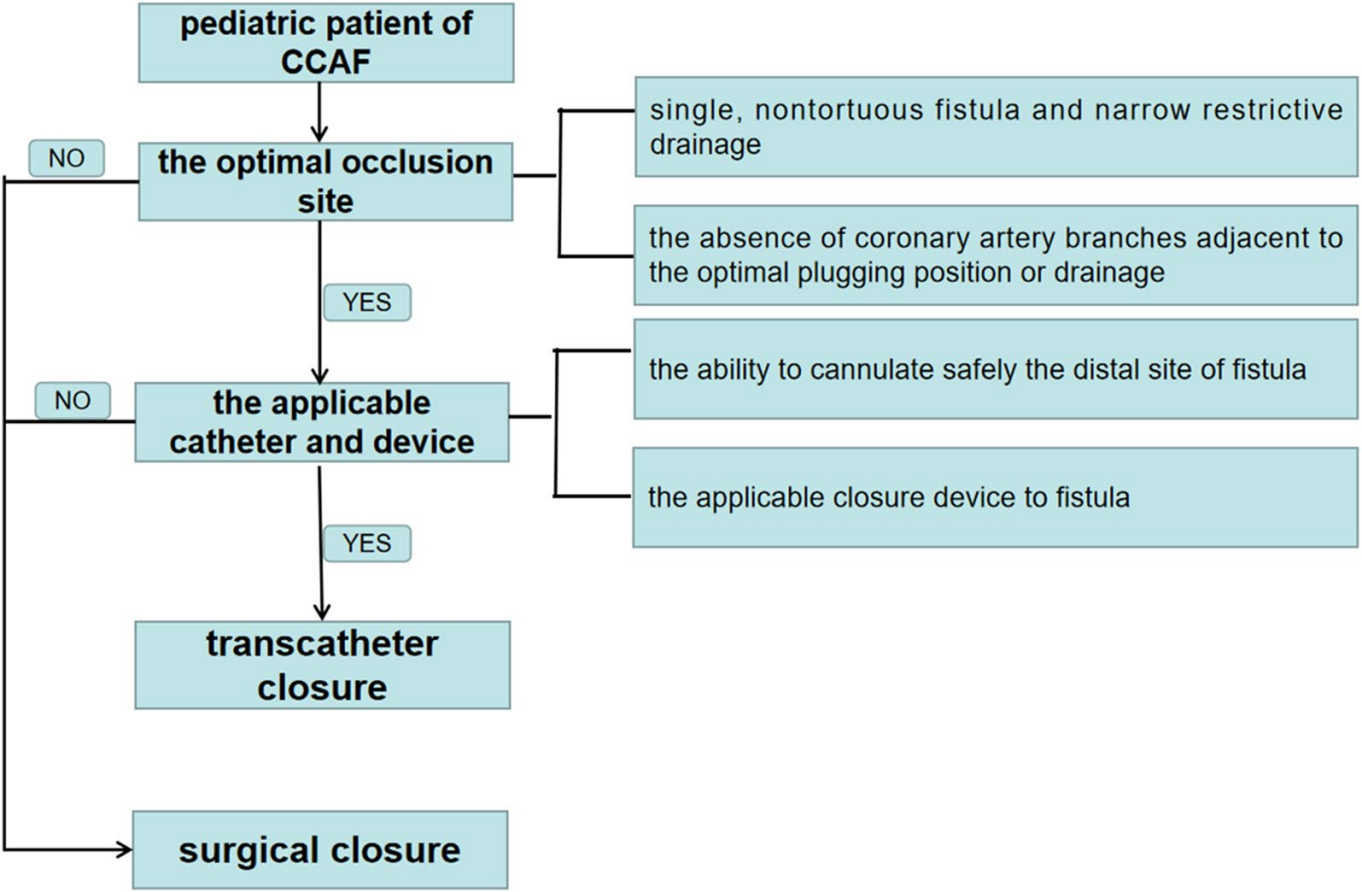
The criterion for surgical or transcatheter closure of CCAF

The indications of surgical closure and transcatheter closure in pediatric patients is controversial and has not been done systematically. The American Heart Association guideline (2011) in adults suggested that transcatheter occlusion should be considered in CCAF patients (class I) [10]. However, the guideline recommendation for the pediatric CCAF population is unavailable. With increasing risk of the developing complications (e.g. congestive heart failure and coronary artery thrombosis, et al.) as growing up [2, 5, 22], fistula closure is now commonly performed before clinical symptoms occurrence. While as for isolated congenial coronary fistula, the indications of transcatheter depended on two factors in our medical center: the optimal occlusion site and the applicable catheter and device (Fig. 3). Results from prior studies indicate that TCC was beneficial to reduce the risk of developing severe complications [5, 12, 23–25]. However, few studies have compared the efficacy and safety between TCC and SC for CCAF therapy.

This study shows that TCC entails shorter hospitalization time and intensive care unit stays, and fewer intra-operative blood loss. The major complications analysis was adjusted for confounding variables by Cox proportional hazards regression models. The fistula drainage (left atrium) was adjusted because it was a significant risk factor for complications after CCAF closure in previous research [23] and its p-value was < 0.1 in our univariate analysis. The intervention method was the only risk factor in this study. After adjusted for the fistula anatomy, compared to SC, TCC was associated with higher risk of major complications (HR: 3.272, P = 0.013).

TCC had a total of 9 (15.3%) re-intervention cases during hospitalization and follow-up when there was zero in SC group. Three prior studies also reported a similar or higher failure rate of TCC for CCAF therapy, with a rate of 13.0%, 16.7% and 25.0% respectively [26–28]. Several factors might influence the success of transcatheter closure, such as a large fistula with high flow rate, multiple fistulous branches, inability to pass a catheter through channels, a long and tortuous coronary artery, and an adjacent artery at high risk of ischemia [29, 30].

In our study, the reasons for re-intervention included myocardial ischemia, incomplete occlusion, occluder embolization and valves injury. Of note, myocardial ischemia was the main reason, which was caused by occluder displacement, coronary artery thrombosis or postoperative coronary artery spasm. Therefore, monitoring electrocardiogram changes at first 24–48 h post-TCC is important to identify this severe complication timely. And careful evaluation of coronary artery anatomical structure may be also helpful to select suitable cases for TCC therapy so as to prevent this complication. In addition, we observed that 5(9.1%) patients in SC group developed coronary artery thrombosis, and whether anti-platelet therapy should be used to these patients needs to be further investigated. Notably, among the TCC groups, during the study period, 4 different kinds of devices were used to manage the fistula. One might argue that the outcomes of patients might be related to the selection of different devices. However, since the number of patients managed with each kind of device were small and there was insufficient power to compare the outcomes between these devices.

Our data also showed that TCC had a higher rate of residual shunt. At follow-up, 10 (6.9%) patients in TCC group had a residual shunt versus 2 (3.6%) patients in SC group, suggesting that SC had a better long-term efficacy, as was better long-term safety. Our results showed that no patients in SC group had moderate or severe valve regurgitation, while 7 (11.9%) patients in TCC group had new onset or worsening of valve regurgitation, which might be due to wire loop caused cusps injury or valve geometry distortion.

### Study limitations

Angiograms were available for review in only 87.6% of the patients studied, and there were no standard methods to evaluate the length and tortuosity of the fistula, therefore, the angiographic features of fistula length and degree of tortuosity were not included in the current analyses. This is a single-center retrospective study, where there is potential for inherent bias such as missing data, referral and selection bias. Our current study has recruited a relatively larger sample of pediatric CCAF patients undergoing fistula closure, however, findings from our current report still needs to be corroborated in prospective and multi-center study in the future.

## Conclusions

Transcatheter closure has less invasive and faster recovery. However, our study suggests that compared to transcatheter closure, surgical closure has higher successful rate and lower risk of complications in pediatric CCAF patients.

## Data Availability

The datasets used and/or analysed during the current study were available from the corresponding author on reasonable request.

## Abbreviations

CCAF: Congenital coronary artery fistula
SC: Surgical closure
TCC: Transcatheter closure
TTE: Transthoracic echocardiography
CCTA: Coronary computed tomographic angiography
SCAG: Selective coronary angiography
HR: Hazard ratios
CI: Confidence intervals
